# Pathological Mechanisms and Potential Therapeutic Targets of Pulmonary Arterial Hypertension: A Review

**DOI:** 10.14336/AD.2020.0111

**Published:** 2020-12-01

**Authors:** Ying Xiao, Pei-Pei Chen, Rui-Lin Zhou, Yang Zhang, Zhuang Tian, Shu-Yang Zhang

**Affiliations:** ^1^Department of Cardiology, Peking Union Medical College Hospital, Chinese Academy of Medical Sciences and Peking Union Medical College, Beijing, China; ^2^School of Medicine, Tsinghua University, Beijing 100084, China

**Keywords:** pulmonary arterial hypertension, right ventricular dysfunction, hemodynamics, therapy advances

## Abstract

Pulmonary arterial hypertension (PAH) is a progressive cardiovascular disease characterized by pulmonary vasculature reconstruction and right ventricular dysfunction. The mortality rate of PAH remains high, although multiple therapeutic strategies have been implemented in clinical practice. These drugs mainly target the endothelin-1, prostacyclin and nitric oxide pathways. Management for PAH treatment includes improving symptoms, enhancing quality of life, and extending survival rate. Existing drugs developed to treat the disease have resulted in enormous economic and healthcare liabilities. The estimated cost for advanced PAH has exceeded $200,000 per year. The pathogenesis of PAH is associated with numerous molecular processes. It mainly includes germline mutation, inflammation, dysfunction of pulmonary arterial endothelial cells, epigenetic modifications, DNA damage, metabolic dysfunction, sex hormone imbalance, and oxidative stress, among others. Findings based on the pathobiology of PAH may have promising therapeutic outcomes. Hence, faced with the challenges of increasing healthcare demands, in this review, we attempted to explore the pathological mechanisms and alternative therapeutic targets, including other auxiliary devices or interventional therapies, in PAH. The article will discuss the potential therapies of PAH in detail, which may require further investigation before implementation.

## 1. Introduction

Pulmonary hypertension (PH) is a cardio-respiratory disease characterized by a resting mean pulmonary arterial pressure (mPAP) ≥ 25 mmHg, as measured by a right heart catheter (RHC). The World Health Organization (WHO) has grouped PH into 5 groups and PAH is class I with pulmonary artery wedge pressure (PAWP) ≤ 15 mmHg and pulmonary vascular resistance (PVR) > 3.0 Woods units. It is a rare and severe condition, with no effective therapeutic option, accompanied by pulmonary arteriole vascular remodeling, luminal stenosis, endothelial dysfunction, and *in situ* thrombosis [1]. It also causes pulmonary vasculature reconstruction, which leads to right ventricular failure and is the leading cause of death. Epidemiological studies [2] have shown that the 5-year overall survival rate of patients with PAH is only 59%. However, data indicate that many patients with PAH and right ventricular failure will die within 2 to 3 years after diagnosis if left untreated. Accurate diagnosis and classification are key to the overall survival rate. However, the pathogenic mechanism of PAH has not yet been elucidated.

Over the past 20 years, new drugs have promoted innovation in PAH therapy. Strategies targeting crucial pathways, including the endothelin-1 (ET-1), prostacyclin, and nitric oxide (NO) pathways, have been established and are widely used in clinical practice. Other adjuvant therapies, including iron supplementation, oxygen inhalation, and anticoagulation, require further evidence to demonstrate their utility in PAH therapy. Multiple prospective cohort studies have found that short-term prognosis has significantly improved, but mortality is still high, and long-term outcomes are uncertain. The aim is to explore the underlying molecular mechanisms and seek other novel therapeutic targets to improve the quality of existing therapeutic approaches. More recently, the 6^th^ World Symposium On Pulmonary Hypertension (WSPH), held in 2018 in Nice, France, reiterated the classification in 5 distinct groups of pulmonary hypertension based on different pathologies and etiological factors [3]. This review mainly focuses on class I PAH.

## 2. Epidemiology and clinical symptoms

PAH is one of five categories of pulmonary hypertension defined by a mean pulmonary arterial pressure (mPAP) ≥ 25 mmHg at rest, a normal capillary wedge pressure ≤ 15 mmHg, and a pulmonary vascular resistance (PVR) > 3 Wood units [2]. PAH is associated with many predisposing factors and is a highly heterogeneous condition, with the etiology varying among races and districts. PAH may occur in the absence of known genetic mutations or other comorbidities — referred to as idiopathic PAH (IPAH) — or be caused by mutations, which is referred to as heritable PAH (HPAH). PAH is also associated with connective tissue disease (CTD-PAH) [4], HIV infection, and drug and toxin usage (fenfluramine, anorexigens, methamphetamine, etc.). PAH may also be associated with pulmonary veno-occlusive disease or capillary hemangiomatosis (PVOD/PCH) and congenital heart disease (CHD-PAH). The prevalence of PAH is approximately 15-60 per million individuals, with an incidence of 5-10 per million individuals per year [5]. IPAH accounts for 35-67% of PAH cases, with an estimated incidence of 1.1-7.6 cases per million individuals according to the US and EU national registries [6]. The French Pulmonary Hypertension Registry [7] indicated that 51 patients with systemic lupus erythematosus (SLE)-associated PAH had an overall 5-year survival rate of 83.9% after diagnosis of the disease. The delay between SLE diagnosis and PAH diagnosis was 4.9 years (range 2.8-12.9 years). The 3- and 5-year overall survival rates were 89.4% (95% CI, 76.2%-96.5%) and 83.9% (95% CI, 68.8%-92.1%), respectively. Anti-SSA/SSB antibodies may be a risk factor for PAH, and the presence of anti-U1-RNP antibodies appears to be a protective factor that influences survival.

The common clinical symptoms of pulmonary hypertension mainly include chest tightness, shortness of breath, and decreased tolerance for progressive activities. As the disease progresses, right ventricular hypertrophy and RV fibrosis develop, accompanied by impaired diastolic function and congestion of systemic circulation. Dyspnea, edema and syncope complications influence the progressive decline in right heart function, causing right heart failure (RHF). The early symptoms may not be typical and are often ignored by patients until right ventricle decompensation. Timely identification of RHF and risk stratification are essential for prognosis and avoidance of lung transplantation in end-stage PAH.

The average survival of PAH patients is approximately 2.8 years in the absence of an effective treatment after diagnosis [8]. The US Food and Drug Administration (FDA) has approved 14 drugs for PAH treatment, mainly targeting pulmonary vasculature vasoconstriction. Endothelin receptor antagonists (ERAs), prostacyclin analogues (PCAs), phosphor-diesterase 5 inhibitors (PDE-Is), and soluble guanylate cyclase stimulators (sGCs) with diverse drug administration routes have been approved in PAH therapy. The French Pulmonary Hypertension Registry [9] collected 674 PAH patients from 17 French centers from October 2002 to October 2003 and found that the 1-year, 2-year, and 3-year survival rates were 87%, 76%, and 67%, respectively. Despite the emergence of vasodilator therapies, PAH remains incurable, and mortality rates are still high. Research has focused on identifying novel therapeutic drugs encompassing broader aspects of the pathophysiology of PAH to improve its prognosis.

PAH remains an incurable and costly disease with a high mortality rate. Accurate diagnosis of PAH is critical for targeted therapy [10], which may require the involvement of PAH centers [11]. The therapeutic regime varies depending upon the distinct risk classification. In newly diagnosed patients, initial combination therapy with ambrisentan and tadalafil is recommended over monotherapy to achieve the best therapeutic outcome[12]. Intravenous administration of prostacyclin is the only therapy that reduces PAH mortality. Advances in diagnostic algorithms and guidelines for risk groups are imminent owing to the reduction in screening time from symptom onset to diagnosis, the predominant determining factor in prognosis. Validated integration algorithms will be essential for diagnosing PAH in the near future [13].

## 3. Pathological mechanisms

### 3.1 Gene mutations associated with PAH

PAH is a genetically heterogeneous disease characterized by vascular remodeling primarily affecting the small pulmonary arterioles (< 100 mm). Remodeling of the pulmonary vasculature involves the obliteration of pulmonary arterioles, muscularization of peripheral arteries and formation of plexiform lesions, which cause the progressive narrowing of blood vessels. There are more than 10 diseases causing genetic mutations that have been linked to the development of PAH, including bone morphogenetic protein receptor (BMPR2), SMAD family member 9 (SMAD9), activin A receptor-like type 1 (ACVRL1), endoglin (ENG), and eukaryotic translation initiation factor 2 alpha kinase 4 (EIF2AK4).

*BMPR2*, a member of the TGF-β superfamily, belongs to the BMP/TGF signaling pathway that induces osteogenesis and cell differentiation. It is the most important pathogenic gene of PAH. More recently, other rare causal candidate genes have been discovered. Whole exon sequencing (WES) screening demonstrated that *T-box 4 (TBX4)* [14], a gene linked to small patella syndrome 8, is the most commonly mutated gene in pediatric patients with HPAH along with BMPR2. Table 1 lists the latest variants associated with PAH mutations.

**Table 1:** Gene variants associated with pulmonary arterial hypertension.

Gene	Gene ID	Chromosome	Disease	Function	Name	Refs
*BMPR2*	659	2q33.1-q33.2	IPAH HPAH	Member of the TGF-β receptor family	Bone morphogenetic protein receptor type 2	[29] [19]
*ACVRL1*	94	12q13.13	HHT/PAH HPAH	Receptor for the TGF-β superfamily	Activin A receptor-like type 1 (ALK1)	[116] [117]
*ENG*	2022	9q34.11	HHT/PAH	Coreceptor of the TGF-β family	Endoglin	[118]
*SMAD9*	4093	13q13.3	HPAH	Transduces signals from the TGF-β family	SMAD family member 9	[119]
*KCNK3*	3777	2p23.3	HPAH IPAH	Encodes the TASK-1 channel, contributes to the membrane potential	Potassium two-pore domain channel subfamily K member 3	[120]
*EIF2AK4*	440275	15q15.1	PVOD/PCH	Phosphorylates eukaryotic translation initiation factor-2 (EIF2)	Eukaryotic translation initiation factor 2 alpha kinase 4	[121]
*TBX4*	9496	17q23.2	Small patella syndrome, PAH in children	Involved in the development of lung disease	T-box 4	[14] [122]
*BMP9*	2658	10q11.22	HPAH IPAH	Binds the TGF-β receptor	Bone morphogenetic protein 9 or growth differentiation factor 2	[15]

IPAH: idiopathic pulmonary arterial hypertension, HPAH: heritable pulmonary arterial hypertension, HHT: hereditary hemorrhagic telangiectasia, PVOD/PCH: pulmonary veno-occlusive disease and/or pulmonary capillary hemangiomatosis.

Recent reports suggest that mutations in *BMP9* may be a newly identified cause of PAH. *BMP9*, also known as growth and differentiation factor 2, is predominantly expressed in the liver and is constitutively secreted into the bloodstream. By binding to ACVRL1 (type I BMP receptor) and BMPR2, BMP9 plays an important role in regulating vascular function and angiogenesis. Gräf et al. found *BMP9* gene mutations in IPAH/HPAH patients among a European Caucasian population through GWES screening. Moreover, Jing *et al*. [15] identified a new pathogenic gene for PAH. It was found in 6.7% of IPAH cases in the Chinese population, where the incidence of IPAH has increased 22-fold. Using recombinant *BMP9* could alleviate hemodynamics in a *BMPR2*-deficient PAH mouse model [16]. Emerging evidence suggests that *BMP9* functions to improve cardiac fibrosis in heart failure [17]; *BMP9*-knockout mice exhibited normal cardiac development. However, one study found that a *BMP9* ^(-/-)^ transgenic model and a *BMP9* inhibitor reduced right ventricular hypertrophy and distal muscularization of pulmonary arteries and reversed the hemodynamic parameters of hypoxic-PH mice [18]. Meanwhile, a neutralizing anti-*BMP9* antibody reduced the proliferation of pulmonary vascular cells and inflammatory cell infiltration in PH mice and protected the PH group. The above results require careful analysis.

#### 3.1.1 *BMPR2* mutations and the TGF-β signaling pathway

*BMPR2* mutations increase the susceptibility to PAH. The BMPR2 protein mainly consists of 3 structures, including the ligand-binding domain, the serine-threonine kinase domain, and the cytoplasmic tail. A meta-analysis of 1,550 PAH patients [19] found that those with *BMPR2* mutations were younger and had a more severe form of PAH, associated with high mortality and poor prognosis, than those with normal *BMPR2*. *BMPR2* haplo-insufficiency is associated with increased endothelial apoptosis, transformational EC metabolism, and PASMC proliferation, which facilitate the development of pulmonary hypertension. *BMPs*, especially *BMP-6* [20], could regulate the proliferation of lymphocytes such as B cells, T cells, and macrophages through *BMPR2*. Downregulation of BMPR2 signaling deleteriously affects granulocyte macrophage colony-stimulating factor (GM-CSF) translation and immune cell recruitment to aggravate PH [21]. In addition, *BMPR2* loss of function enhances inflammation in fibroblasts [22].

Van der Bruggen *et al*. [23] demonstrated that individuals with PAH and *BMPR2* mutations have worse right ventricular function than those with PAH and normal *BMPR2*. In addition, animal studies found that [24] *BMPR2*-mutant mice showed decreased exercise capacity and poor response to stress load and that myocardial hypertrophy in animals was accompanied by right ventricular energy metabolism disorder.

How does *BMPR2* affect the pathogenesis and disease progression of PAH? Familial PAH (FPAH) is considered to be an autosomal dominant disease, and 70% of patients afflicted with the disease exhibit *BMPR2* germline mutations [25]. Twenty-five percent of IPAH cases are associated with *BMPR2* gene mutations. Among patients with a family history of PAH, 58-74% have *BMPR2* mutations, which are present in 3.5-40% of patients with IPAH [26].

Almost 80% of *BMPR2* mutation carriers do not necessarily progress to PAH [27], highlighting their incomplete penetrance. With this background, there are significant sex differences. The penetrance rate of *BMPR2* mutations in women is 42%, compared with 14% in men [28]. Therefore, the hereditary, environmental and epigenetic regulatory mechanisms that cause BMPR2 dysfunction are implicated in disease pathogenesis. There are more than 144 distinct *BMPR2* mutations that inhibit protein function found in PAH patients [29]. The possible mechanisms of BMPR2 degradation include the lysosomal degradation pathway and the TNF-α-mediated ubiquitination process. Drugs that increase BMPR2 expression and degradation have been exploited for preclinical trials [30].

**Figure 1.**
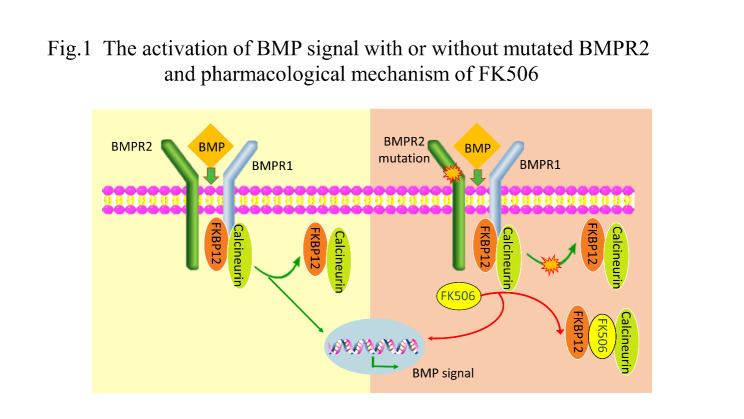
Activation of BMP signaling with or without mutated BMPR2 and the pharmacological mechanism of FK506. BMP signaling in the presence of normal or mutated dysfunctional *BMPR2*. Mutated *BMPR2* protein disturbs the dissociation of FKBP12-calcineurin from *BMPR1* when stimulated by activating doses of BMPs. FK506 binds to FKBP12 and promotes the dissociation of FKBP12-calcineurin from BMPR type 1 receptors and then activates the downstream signaling pathway. BMP: bone morphogenetic protein; FKBP12: FK506-binding protein 12.

#### 3.1.2 FK506

FK506 (tacrolimus), a calcineurin inhibitor and a BMPR2 activator, is the binding partner of FKBP12, which is a 12-kDa FK506-binding protein and pharmacological target of FK506. Spiekerkoetter et al. [31] reported that a low dose of FK506 (tacrolimus, 15 ng/ml) could reverse occlusive vasculopathy in MCT and sugen-hypoxia-induced PAH rat models and inhibit the suppression of the BMPR2 signaling pathway. FK506 is an immune-suppressant widely used as an ointment in the treatment of atopic dermatitis.

The *BMPR2* mutation impairs the function and prevents the dissociation of FKBP12-calcineurin from BMPR type 1 receptors (ALK1, ALK2, and ALK3) when exposed to the subactivating ligand of BMPs. FK506 binds to FKBP12, causing its detachment from the BMPR type 1 receptors, thus activating BMPR2-mediated signaling. Interestingly, a previous study [32] also showed that high doses of FK506 (>10-fold higher than the dose of the above study) induced systemic hypertension and nephrotoxicity by enhancing ET-1 production and inhibiting endothelial nitric oxide synthase (eNOS) activity in mouse models. Fig. 1 depicts the activation of the BMP signal with or without mutated *BMPR2* and the pharmacological mechanism of FK506.

**Table 2:** Clinical trials and potential therapeutic targets in pulmonary arterial hypertension.

Drug/agent	Mechanism	ClinicalTrials.gov identifier	Participants	Study design	Study duration	Primary outcome measure	Outcome
Tacrolimus (FK506)	Activator of BMP signaling	NCT01647945	23 patients with PAH	Single center, phase II randomized, placebo-controlled study	16 weeks	Safety of low-dose FK-506 in PAH	Completed
Tocilizumab	Humanized anti-IL6R antibody	NCT02676947	29 patients with group 1 PAH	Open-label phase II trial	6 months	Safety in terms of the incidence and severity of adverse events	Completed
Anakinra	Recombinant IL-1 receptor antagonist	NCT03057028	6 patients with stable PAH and RV failure	Single-arm, open-label, phase IB/II pilot study	14 days	Change in exercise capacity as determined by peak oxygen consumption and ventilatory efficiency	Completed
Rituximab	Anti-CD20 antibody	NCT01086540	SSc-PAH	Double-blind, placebo-controlled, phase II, multicenter, randomized trial	48 weeks	Change from baseline in 6MWD	Active, not recruiting
Dichloroacetic acid (DCA)	Inhibition of pyruvate dehydrogenase kinase	NCT01083524	20 adult patients with IPAH	Phase I, open-label, two-center study	28 weeks	Safety and tolerability of DCA	Completed
Apabetalone (RVX-208)	BET inhibitor	NCT03655704	Estimated 10 participants	Early phase I, two-center, open-label trial	16 weeks	Change in PVR	Recruiting
Olaparib	PARP1 inhibitor	NCT03251872	Estimated 6 participants	Open-label, early phase I trial	16 weeks	Change in PVR	Recruiting
Anastrozole	Estrogen inhibitor	NCT01545336	18 participants	Double-blind, placebo-controlled, phase II study	3 months	Plasma estradiol (E2) level, tricuspid annular plane systolic excursion (TAPSE)	Completed
Metformin	Multifunctional aromatase inhibitor and AMPK activator	NCT03617458	160 participants	Phase II, 2×2 factorial, randomized, blinded trial	12 weeks	Change from baseline in 6MWD	Recruiting
Imatinib	Selective tyrosine kinase inhibitor	NCT01392495	17 participants	Open-label, phase III, nonrandomized trial	144 weeks	Number of patients with adverse event and deaths	Terminated for severe adverse effects
Dimethyl fumarate	Nuclear factor erythroid 2-related factor 2 (Nrf2) activator	NCT02981082	34 participants with SSc-PAH	Double-blinded, phase I, placebo-controlled pilot study	24 weeks	Improvement in 6MWD	Recruiting
Bardoxolone methyl	Nrf2 pathway-activating agent	NCT02657356	202 participants with CTD-PAH	Phase III, double-blind, randomized, placebo-controlled trial	24 weeks	Change from baseline in 6MWD	Not recruiting
Gene-enhanced EPCs (PHACeT trial)	Cell therapy	NCT00469027	7 participants with PAH	Phase I, open-label, dose-escalation study	5 years	Tolerability and safety of the injection of genetically engineered progenitor cells	Completed
Pulmonary artery denervation (PADN)	Inhibitor of sympathetic stimulation	NCT02284737	Estimated 270 participants	Phase IV, prospective, multicenter, randomized control trial	6 months	PAH-related events, death including lung transplantation, atrial septostomy, worsening of PAH	Recruiting

DCA: dichloroacetic acid; DMF: dimethyl fumarate; Nrf2: nuclear factor erythroid 2-related factor 2; PADN: pulmonary artery denervation; PAH: pulmonary arterial hypertension; 6MWD: six-minute walk distance; SSc-PAH: systemic sclerosis with pulmonary arterial hypertension; EPCs: endothelial progenitor cells; IPAH: idiopathic pulmonary arterial hypertension; CTD-PAH: pulmonary arterial hypertension associated with connective tissue disease.

Furthermore, a single center, 16-week, phase IIa RCT ensured the safety and tolerability of FK506. It revealed that [33] a low dose of FK506 could improve endothelial dysfunction and reverse pulmonary hypertension in stable PAH patients with great tolerance. However, the improvements in six-minute walk distance (6MWD) and heart failure were not significant when compared to adverse effects such as nausea and diarrhea (NCT01647945, Table 2). The results related to FK506 shed some light on drug therapies based on the pathogenesis of PAH. Toxicity should also be accounted for before starting clinical studies.

### 3.2 Apoptosis of PAECs and proliferation of PASMCs

PAH is characterized by apoptosis resistance phenotypes of pulmonary arterial endothelial cells (PAECs) and enhanced proliferation of pulmonary arterial smooth muscle cells (PASMCs) from the perspective of pathological insights, which drive the progression of PAH. However, the underlying mechanisms remain unclear, and a novel treatment option is desperately needed to combat the progression of disease. PAH-SMCs featured increased production of inflammatory factors and adaptation of mitochondrial metabolism to a hyperproliferative state, similar to cancer cells, suggesting that PAH-SMCs are different from SMCs isolated from healthy individuals. Vascular remodeling and muscularization of distal pulmonary arterioles resulted in right ventricular failure, causing death. Finding novel treatments that reverse pulmonary vascular remodeling remains an urgent need.

Drugs that target PASMC proliferation may shed new light on novel treatment strategies. Investigation into the molecular mechanisms of PASMCs revealed the pharmaceutical effects of celastramycin in PAH [34] via high-throughput screening. Celastramycin, a benzoyl pyrrole-type compound, ameliorates pulmonary hypertension in three experimental PAH animal models by reducing excessive proliferation of PASMCs, leading to less inflammation and cytosolic reactive oxygen species (ROS) levels, as well as recovering mitochondrial energy metabolism. It may be a promising potential drug for PAH.

Evidence has shown that *in vivo* pharmacological activation of KCNK3 channels has beneficial effects in monocrotaline-induced PH in rats. Furthermore, inhibition of voltage-gated potassium channels could represent one potential mechanism involved in some drug-induced PH.

In addition, PAECs have been strongly implicated in PAH pathogenesis because the existing drugs mainly target endothelium vasodilatation-associated pathways. Recent preclinical studies with PASMC/PAEC-specific target gene knockout mice are the ideal tool for research on vascular endothelial injury at the cellular or animal level.

### 3.3 Inflammation in PAH

The inflammatory signaling pathway may also be a key factor in the pathologic progression of PAH, particularly in terms of associated PAH. Although we have recognized this phenomenon for many decades, the understanding of inflammatory molecules in PAH is still limited. Epidemiological studies [35, 36] and an experimental research study [37] have found that PAH is associated with immune dysregulation and abnormal B cell, NK lymphocyte, and regulatory T cell (Treg) activity. Innate immune system dysfunction is associated with plexiform lesions of PAH. This finding suggests that inflammation and autoimmune mechanisms are closely related to the prognosis and survival of PAH patients.

Histopathology studies of PAH patient lungs support the hypothesis of in?ammation as a powerful pathological driver of PAH. PAH patients generally exhibit signs of chronic inflammation even without an immune-related condition, most prominently delineated by elevated circulating cytokine levels and perivascular inflammatory infiltrates [38, 39]. In addition, PAH can present autoimmune features such as elevated anti-nuclear, anti-fibroblast, and anti-endothelial cell antibodies. The pathological process is accompanied by the accumulation of perivascular inflammatory cells, such as macrophages, dendritic cells, mast cells and T and B lymphocytes, which are mainly distributed in the submucosa around remodeling pulmonary vessels. Furthermore, dysfunction of BMPR2 in fibroblasts can cause elevated inflammation. Because the present treatments are not curative, the clinical management of patients primarily focuses on achieving a symptom-free condition, aiming to improve quality of life.

Inflammatory cells aggregate around the vascular walls and elevate peripheral blood inflammatory cells simultaneously. The levels of various pro-inflammatory cytokines in the blood are associated with the survival rate of patients. Moreover, some research has discovered impairment of cellular immunity (T lymphocytes, natural killer (NK) cells, and macrophages) in patients with PAH [40-42]. NK cells are being identified as important regulators of vascular remodeling in different physiological and pathological conditions. NK cells isolated from PAH patients exhibit an impaired phenotype with high responsiveness to transforming growth factor-β (TGF-β), accompanied by reduced disease-associated killer immunoglobulin-like receptors and matrix metalloproteinase 9 production [41]. Additionally, CD4+ CD25+ Foxp3+ regulatory T-cells (Tregs), identified as a key type of potent immunomodulator of the adaptive immune system, have also been reported to be important in PAH [43]. Athymic nude rats lacking Tregs showed exaggerated lung vascular injury, macrophage recruitment, and elevated pulmonary pressure induced by vascular endothelial growth factor receptor 2 (VEGFR2) blockade [44]. Many retrospective and prospective analyses have found that patients with PAH have elevated levels of the cytokines interleukin-1β, IL-6, and IL-8 and the chemokine CCL2/MCP-1, among others [45, 46]. Moreover, the increased circulating concentrations of certain pleiotropic cytokines and inflammatory substances cause a regulatory immune response through canonical and noncanonical signal transduction pathways.

It has been shown that inflammation may play a significant role in the prognosis and treatment response in CTD-PAH. Patients with a simultaneous diagnosis of PAH and a CTD, including SLE (systemic lupus erythematosus), MCTD (mixed connective tissue disease), and primary SS (Sjögren's syndrome), tend to benefit from receiving first-line intensive immunosuppressive regimens, which are accompanied by better cumulative rates free of PAH-related death as well as better short-term and long-term outcomes [47]. Sun et al. [48] demonstrated two distinct inflammatory phenotype clusters of SLE-PAH, the vasculopathic and vasculitic subtypes. The vasculitic subtype shows systemic manifestations, high SLE disease activity, and a highly inflammatory phenotype. A score was calculated by combining the time interval between the diagnosis of SLE and PAH and the Systemic Lupus Erythematosus Disease Activity Index (SLEDAI) score and based on the clinical phenotypes associated with the weighted score. The vasculitic subtype had the desirable 3-year treatment goal achievement (TGA) rate of 71.2%, compared with the 60.7% TGA rate in the vasculopathic subtype (p = 0.31 HR 1.34, 95% CI 0.76 to 2.31). The mortality and prognosis were not worse than those of the vasculopathic subtype [49]. This evidence indicates that PAH patients with a stronger inflammatory profile may have a better treatment response to immunosuppressant therapy than those with a weaker inflammatory profile. However, no definitive conclusion can be made before clinical trials to eliminate the influence of comorbidities, heterogeneity of disease, and other confounding factors. Treatment protocols for SLE-PAH should be scrutinized on account of the high mortality associated with this disease.

It remains unknown whether inflammation is the cause of pulmonary vascular remodeling or is nothing but the consequence of the pulmonary high-pressure microenvironment. Novel drugs with anti-inflammatory or immunomodulatory properties may be potent therapeutic targets but are still obscure given that many patients fail to respond to broad immunosuppressive therapy with corticosteroids. The following is a list of the different biological effects and possible mechanisms targeting different inflammatory factors and immune profiles in PAH.

#### 3.3.1 IL-6

IL-6 is a multifunctional pro-inflammatory cytokine that is elevated in the serum of patients with PAH and can be used to predict the survival rate of IPAH. Hypoxic signaling likely contributes to the inflammatory milieu of PH as well. IL-6 plays an important role in immune regulation as well as metabolic, proliferative, and regenerative processes in the body. A study [50] showed that transgenic mice with lung tissue-specific IL-6 overexpression featured spontaneous PH and pulmonary vascular occlusion at normal oxygen partial pressure, whereas hypoxia aggravated the severity of PH. In addition, mice with specific knockout of IL-6R exhibit resistance to hypoxia-induced pulmonary hypertension (HPH). Further animal experiments revealed that IL-6 continuously activated the inflammatory response by activating the FGF pathway, stimulating apoptosis of ECs and PASMC proliferation, and disrupting the BMPR2 signaling pathway.

Clinical studies have reported [51, 52] that high levels of inflammatory mediators are predictive of survival rate and that the severity of right ventricular failure is associated with worse outcomes. Animal studies provide further evidence regarding the usefulness of immunotherapy in PAH. Experiments have confirmed that [53] a recombinant IL-6 receptor antagonist can reverse pulmonary artery remodeling in experimental PAH models and protect heart function. Patients with IPAH and HPAH had increased IL-6 levels in serum and lung dissection samples along with autoimmune disorders, which are associated with an increased risk of death. PAH-PASMCs showed upregulated expression of IL-6R, with IL-6 appearing to signal both via the membrane-bound (classic or cis) pathway and the trans-signaling pathway (gp130). Tocilizumab, a humanized anti-IL6R monoclonal antibody approved for rheumatoid arthritis, disturbs both classical and trans-signaling and improved the symptoms in a patient with mixed connective tissue disease and severe refractory PAH [54]. An open-label phase II trial was performed to evaluate the safety and efficacy of tocilizumab in 29 patients with class I PAH (ClinicalTrials.gov identifier: NCT02676947). Inhibiting all IL-6 signaling might exert severe adverse effects because IL-6 plays a vital role in the physiological functioning of the body. Evidence suggests that [55] patients with weakened adaptive immunity respond positively and benefit more from IL-6 antagonist therapy than patients with normal adaptive immunity. Thus, it is important to identify the patients who would benefit most from IL6R-inhibiting treatment [56].

#### 3.3.2 IL-1

The interleukin-1 (IL-1) family comprises three ligands: IL-1α, IL-1β, and IL-1 receptor antagonist (IL-1Ra). Elevated levels of IL-1 have been implicated in human PAH diseases [52, 57]. IL-1 receptor antagonist (IL-1Ra) was shown to prevent the development of pulmonary hypertension in monocrotaline-induced rats more than two decades ago, although this has never been investigated in humans with PAH. Anakinra, an IL-1Ra, is currently U.S. FDA-approved for use in rheumatoid arthritis, Castleman’s disease, and juvenile arthritis but has also demonstrated efficacy in a wide array of inflammatory conditions. An expanding role for IL-1 is emerging with the development of biomedicine.

IL-1α and IL-1β are synthesized by monocytes, macrophages, neutrophils, hepatocytes, and other cells *in vivo*, and their precursor molecules become biologically active when cleaved by the enzyme caspase-1. There are two distinct forms of IL-1R, the activated IL-1RI containing the long cytoplasmic domain and the biologically inert IL-1RII containing a short intracellular domain. IL-1R accessory protein (IL-1RAcP), another IL-1 family member, could interact with the IL-1/IL-1RI combination to form a macromolecular complex by recruiting MyD88 (myeloid differentiation factor 88), IRAK (IL-1R-associated kinase), and TRAF6 (tumor necrosis factor (TNF) receptor-associated factor 6) to activate signal transduction pathways such as the nuclear factor-κB (NF-κB), AP-1 (activator protein-1), JNK (c-Jun N-terminal kinase), and p38 MAPK (mitogen-associated protein kinase) pathways.

The ambiguous function of IL-1 in PAH is attracting increasing interest from both bioscience and medical research alike. Parpaleix et al. [58] revealed that IL-1R1(-/-), MyD88(-/-), and control mice given the IL-1R1 antagonist anakinra are protected against hypoxic pulmonary hypertension and perivascular macrophage recruitment. Interleukin (IL)-1β binds to IL-1R1, thereby recruiting the molecular adaptor MyD88 and inducing IL-1, IL-6 and TNF-α synthesis through NF-κB activation.

Recently, Trankle and colleagues [59] reported the effects of anakinra in PAH in their pilot study. The single-arm, open-label, phase IB/II pilot study found that IL-1 blockade could reduce inflammation in PAH and reverse right venticular failure (RVF). These results call for further exploration of anakinra to address the systemic inflammatory component of PAH. Anakinra reversed pulmonary hypertension partially through IL-1 receptor 1/MyD88 signaling and markedly attenuated pulmonary vessel remodeling in monocrotaline-treated rats. This study evaluated the safety and feasibility of anakinra in the treatment of stable PAH patients with complicated RHF based on standard drug therapy. It was found that anakinra reduced the inflammatory load by reducing serum hs-CRP levels but without significant clinical benefits owing to the limitations of the study. This study provides clear proof of principle as well as safety and feasibility data supporting IL-1 receptor antagonism with anakinra to address the systemic inflammatory conditions of PAH. Conversely, anakinra is emerging as a potent clinical target in the REDHART study [60] (mainly including patients with acute exacerbation of systolic heart failure).

#### 3.3.3 CD-20

Rituximab, an anti-CD20 chimeric monoclonal antibody that targets the B lymphocyte CD20 protein, has been reported to be effective in CTD-PAH and SLE-PAH in case reports [61]. The ongoing phase II randomized clinical trial ASC01 is examining rituximab treatment in SSc-PAH patients (ClinicalTrials.gov identifier: NCT01086540), with a completion date estimated in December 2019. This study mainly focused on 6MWD change at week 24, change in PVR and time to clinical worsening from baseline through week 48. Another animal study [62] also found that targeting IL-17 attenuated hypoxia-induced pulmonary hypertension through downregulation of the β-catenin pathway.

#### 3.3.4 TNF-α

Endogenous TNF-α is secreted by activated human CD8^+^ T cells, causing mitochondrial hyperpolarization, which decreases the K^+^ current, increases the Ca^2+^ current and activates NFAT in normal human PASMCs. TNF-α inhibits PASMC PDH activity *in vivo*. A TNF-α antagonist, etanercept [63], prevents and ameliorates symptoms in MCT-induced rodents by decreasing endogenous prostaglandins, improves apoptotic resistance of PASMCs, and activates angiogenesis and adrenergic signaling. However, no significant improvement in heart function or 6MWD was observed.

### 3.4 Epigenetic modifications

Epigenetics refers to phenotype changes mediated by altered gene expression, not DNA sequence [64]. Epigenetic mechanisms can be acquired and/or heritable, and there are three major mechanisms: (i) methylation of CpG islands mediated by DNA methyltransferases; (ii) histone modification; and (iii) regulation by microRNAs (miRNAs) [65]. Epigenetic perspectives shed new light on how the environment influences gene expression and disease susceptibility in cardiovascular disease. Histone deacetylase (HDAC) dynamically regulates epigenetic programming and is critical for cell proliferation and survival. HDAC inhibitors have been reported to produce opposite effects in different preclinical PAH models [66, 67]. However, little is known about the epigenetic mechanism in PAH. The reversible nature of these epigenetic modifications and the existence of chromatin-modifying enzymes suggest the possibility of epigenetic-based PAH therapy.

BRD4 is a member of the bromodomain and extraterminal (BET) motif family, members of which are critical epigenetic drivers for many cardiovascular diseases. BRD4 can promote cell survival, stimulate proliferation, inhibit apoptosis, and modulate the pro-inflammatory phenotype by increasing the transcriptional expression of the cytokines IL-6 and IL-8. It exerts a critical transcriptional modulator function in the context of accumulating DNA damage.

Studies have shown [68] that the overexpression of BRD4 in PAH is closely associated with the aberrant DNA damage response (DDR) mediated by FoxM1. Recently, a preclinical study indicated that [69] inhibiting BRD4 by using JQ1 and siRNA could enhance pulmonary hemodynamics and alleviate vascular remodeling in sugen5416-hypoxia rats by reducing the apoptosis resistance and proliferation capacity of PAH-SMCs. A new clinical trial with the BRD4 inhibitor apabetalone (RVX-208) in PAH patients is upcoming. The trial number is NCT03655704.

### 3.5 DNA damage and repair

DNA damage may occur when the body encounters endogenous or exogenous genotoxic agents, which results in the impairment of cellular DNA integrity and function. PAH is associated with inflammation and oxidative stress, which could induce DNA damage. DNA damage and repair dysregulation has been known as a strong trigger for PAH progression since the discovery of microsatellite instability and somatic genetic mutations in the PAH lung [70, 71].

A study [72] found 30.2% of PAH-PAECs had chromosomal abnormalities in comparison to 5.3% of the corresponding control cells. Aside from PAECs, peripheral blood mononuclear cells (PBMCs) obtained from patients or their relatives with PAH presented an elevated baseline mutation rate and led to mutagen-induced DNA damage. A study has also found that poly (ADP-ribose) polymerase-1 (PARP-1), a critical enzyme implicated in DNA repair, was overexpressed in human PAH distal pulmonary arteries and cultured PAH-PASMCs [73]. A further animal study showed that the PARP inhibitor veliparib (ABT-888) reduces RV pressure and reverses PAH development as well as RV hypertrophy in sugen-hypoxia- and monocrotaline-induced rat models.

The DNA damage/PARP-1 signaling pathway has the potential to be the next novel therapeutic target for PAH treatment. Another PARP1 inhibitor, olaparib, is in an early phase I clinical trial for PAH (ClinicalTrials.gov identifier: NCT03251872). It is known to inhibit cancer growth and is a safe and effective therapy approved for ovarian cancer in Canada, Europe and the United States. However, whether DNA damage contributes to lung vascular remodeling and predates PAH development remains unknown. Targeting DNA damage or PARP signaling is a novel and promising approach to the development of novel therapies for PAH.

### 3.6 Estrogen signaling

The chemical reaction that converts androgens to estrogen is facilitated by aromatase. Aromatase is hyperactive in the pulmonary vasculature of PAH patients. An epidemiological study has suggested that [74] 70-80% of patients with PAH are female. However, many studies have demonstrated [75, 76] the so-called “estrogen paradox”: in rodents, the estrogen hormone is protective against PAH because of pathological mechanisms that are different from those in humans.

Conversely, male PAH patients exhibit a poorer lung transplant-free survival rate and lower overall survival than female patients [77]. Other studies [78, 79] suggested that high levels of estradiol (E2) and low levels of dehydroepiandrosterone sulfate (DHEA-S) were correlated with poor hemodynamics and thus poor outcome of PAH in postmenopausal women and male PAH patients. The MESA study [80] (Multi-Ethnic Study of Atherosclerosis) demonstrated that men had greater RV mass (≈8%) and larger RV volumes but lower RV ejection fraction (4% in absolute terms) than women. The ability of adaptive remodeling of the right ventricle (RV) may explain the roll of sex in PAH survival, as males have a poorer RVEF response when subjected to an increased afterload than females.

Moreover, a study [81] demonstrated that estrogen downregulates the expression of BMPR2. Estrogen-driven suppression of BMPR2 signaling may make women prone to PAH by contributing to a pro-proliferative phenotype in PASMCs. Recently, a new study [82] found that obesity, which alters estrogen metabolism, could induce PAH by increasing the production of 16α-hydroxyketone (16αOHE1) from visceral adipose tissue, which contributed to oxidative stress. The estrogen inhibitor anastrozole attenuates pulmonary vascular remodeling and increases reactive oxygen species production in the lung in a spontaneous PAH leptin-deficient (ob/ob) mouse model. A small-scale clinical trial tested the estrogen inhibitor anastrozole and found it to be a safe, well-tolerated treatment option for PAH (NCT01545336) [83].

Unfortunately, the functional limitations and reasons for the imbalanced female-to-male ratio in disease prevalence remain unclear. Sex-based differences in sex hormone signaling and its potential beneficial effects are therefore left for future work.

### 3.7 Metabolism dysfunction

PDK inhibits pyruvate from entering the mitochondria and increases lactate dehydrogenase. Dichloroacetic acid (DCA), a PDK inhibitor, partially restores RV structure and function by promoting glucose and mitochondrial oxidation and reducing pyruvate dehydrogenase phosphorylation in PAH rodent models. A 4-month clinical trial has shown [84] that inhibition of PDK (pyruvate dehydrogenase kinase) improves pulmonary hypertension in genetically susceptible patients. The study revealed that PDK inhibition could ameliorate hemodynamic parameters, but outcomes varied, with great heterogeneity in patient response. The advantage of transforming metabolic-targeted drugs into clinical strategies remains to be determined.

A previous study [85] observed that a metabolic shift from mitochondrial oxidative phosphorylation to enhanced aerobic glycolysis in the pathogenesis of PAH. TGF-β/BMPR2 and NOTCH/AMPK-AKT are currently known to be involved in the malignant proliferation of PAH PASMCs. Endothelial AMP-activated protein kinase (AMPK) plays a vital role in cardiovascular homeostasis. The molecular mechanisms underlying this phenomenon remain unclear. Recently, it has been found [86] that alpha-enolase regulates the malignant phenotype of PASMCs via the AMPK-AKT pathway. The results of that study also found that ENO1 levels were enhanced in PAH patients and hypoxic pulmonary hypertension rodent models. Pharmacological inhibition of ENO1 regulates metabolic pathways from mitochondrial respiration to glycolysis and reverses the hypoxia-induced metabolic shift in PASMCs and HPH models. One study shows that endothelial-specific AMPK-knockout (eAMPK^-/-^) mice exhibit accelerated PAH development accompanied by elevated growth factors and enhanced proliferation of PASMCs when exposed to hypoxia [87, 88]. Endothelial AMPK may be a novel protective therapeutic target for the treatment of PAH. Metformin, a multifunctional aromatase inhibitor and AMPK activator, has been shown to slow the exacerbation of PAH in sugen5416-hypoxia (SuHx) rats [89]. A phase II 12-week clinical trial investigating the function of metformin is in progress (NCT03617458). However, other studies uncovered the controversial role of AMPK signaling in PAH [90, 91], suggesting that the preferentially beneficial function of metformin therapy may be restricted to PAH associated with metabolic syndrome.

Imatinib is a selective tyrosine kinase inhibitor (TKI) that inhibits the activation of platelet-derived growth factor receptor (PDGFR), which is highly expressed in patients with PAH and is considered one of the exacerbating factors of disease progression [92, 93]. The IMPRES (Imatinib in Pulmonary Arterial Hypertension) study [94, 95], a randomized efficacy study, found that imatinib could improve exercise capacity and hemodynamics among PAH patients inadequately responsive to 2-3 PAH-specific therapies (ClinicalTrials. gov identifier: NCT01392495). However, the severe adverse effects led to the termination of the clinical trial. Lacking target selectivity brings about significant side effects, such as subdural hematoma, along with a high discontinuation rate, which limits the utility of PAH treatment. Pulmonary veno-occlusive disease and/or pulmonary capillary hemangiomatosis (PVOD/PCH) are categorized as group 1 pulmonary hypertension, with no effective medical therapy except for lung transplantation. Recently, a small observational study [96] found that imatinib improved survival time and enhanced exercise capacity in patients with PVOD/PCH and may function as a bridge therapy to lung transplantation.

It is worth mentioning that dasatinib, another BCR-ABL tyrosine kinase inhibitor, causes pulmonary vascular damage and increased susceptibility to pulmonary hypertension by inducing PAEC apoptosis and mitochondrial ROS production in a dose-dependent manner, whereas imatinib does not [97]. This intriguing phenomenon suggests that studying the metabolism of PAH pathophysiology is useful for understanding its effects and drug toxicity and providing a proof of concept for promising therapeutic options.

### 3.8 Oxidative stress

Drugs targeting oxidative stress for PAH treatment, such as methods targeting apoptosis signal-regulating kinase 1 and mitochondrial ROS production, are being explored [98, 99]. A preclinical study found [100] that an ASK1 inhibitor could halt the progression of pulmonary vascular and RV remodeling in murine PAH models. Nuclear factor erythroid 2-related factor 2 (Nrf2), another redox-regulated transcription factor, plays a key role in the redox homeostatic gene regulatory network. It can increase cytoprotectiveness, induce antioxidant enzymes/proteins, and protect against cardiopulmonary alterations caused by oxidative stress. A study found [101] that Nrf2-deficient transgenic mice developed more deteriorative RVF than WT mice, whereas Nrf2 activators attenuated RVF and pulmonary vascular remodeling.

A recent study found [102] that dimethyl fumarate (DMF), a nuclear factor erythroid 2-related factor 2 (Nrf2) activator, was effective in the sugen-hypoxia experimental PH model. DMF ameliorated the development of pulmonary hypertension and reversed hemodynamic changes in PH mice and exerted its anti-inflammatory effects by reducing oxidative stress and directly targeting the NF-κB signaling pathway through covalent modification of p65. A phase I study among SSc-PAH patients is in progress (ClinicalTrials.gov identifier: NCT02981082). In 2013, the US Food and Drug Administration (FDA) approved DMF for the treatment of relapsing-remitting multiple sclerosis (RRMS) [103, 104].

Another Nrf2 pathway-activating agent, bardoxolone methyl, which was reported to decrease the production of toxic reactive oxygen species (ROS), seems to be effective in PH [105]. Another study [106] identified a novel hybridization with isosorbide 5-mononitrate and bardoxolone methyl that attenuated right ventricular hypertrophy and vascular muscularization in MCT-induced PAH rats. A diverse combination of multiple pathway inhibitions in pulmonary vascular remodeling makes it possible to find novel promising pharmacological therapeutic options. A phase III double-blind, randomized, placebo-controlled trial among CTD-PAH patients is in the patient recruitment stage (ClinicalTrials.gov identifier: NCT02657356).

**Figure 2.**
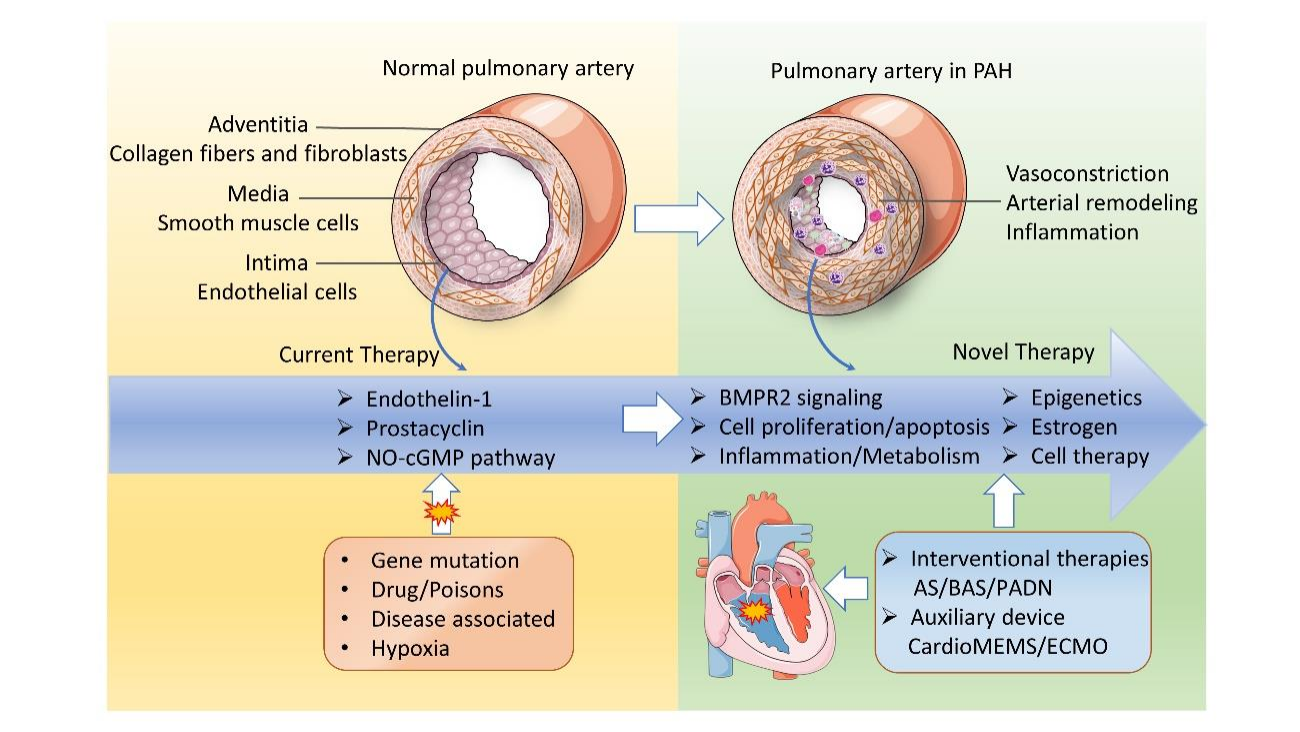
Pathobiology of PAH and potential therapeutic targets. Pathological mechanisms and potential therapeutic targets of PAH. The pulmonary artery wall consists of three structural layers, including the adventitia, media, and intima. Various pathogenic factors, such as gene mutations, drugs/poisons, and hypoxia, can induce pulmonary arteriole vascular vasoconstriction, characterized by luminal stenosis, endothelial dysfunction, inflammation, infiltration, etc., ultimately causing RHF. The endothelin-1, prostacyclin, and nitric oxide pathways have been targeted in clinical practice and are three pivotal pathways approved in PAH management. Potential therapeutic targets are emerging as the pathobiology of PAH is revealed. AS: atrial septostomy; BAS: balloon atrial septostomy; PADN: pulmonary artery denervation; ECMO: extracorporeal membrane oxygenation.

## 4. Other potential therapies

PAH remains a chronic progressive and irreversible disease that eventually results in RVF or death. Lung transplantation is the only final effective option for late-stage PAH and patients classified as New York Heart Association (NYHA) functional class other than combined treatment. Artificial organ support and other potential adjuvant therapy options are pivotal for the treatment strategy as well. Assisting machines and devices have been invested to support the failing right ventricle, including extracorporeal membrane oxygenation (ECMO). Pumpless membrane oxygenators inserted between the pulmonary artery and the pulmonary veins or left atrium (PA-LA) and CardioMEMS are also an alternative therapeutic option [107, 108]. At the same time, it is important to maintain fluid management, supplementary oxygen therapy, and intensive care for PAH patients with RVF. Fig. 2 summarizes the pathobiology of PAH and potential therapeutic targets.

### 4.1 Cell therapy

Induced pluripotent stem cells (iPSCs) from skin fibroblasts of PAH patients provide new insight into the molecular mechanism and reprogramming in *BMPR2* mutation carriers and PAH patients for individualized therapy [109]. The Pulmonary Hypertension and Angiogenic Cell Therapy (PHACeT) trial [110] is a phase I, dose-escalation study investigating the tolerability of endothelial progenitor cells overexpressing endothelial nitric oxide synthase via plasmid DNA in 7 patients with IPAH. In the study, gene-enhanced EPCs were injected into the patients’ blood stream through the central vein via a Swan-Ganz catheter. No improvement in pulmonary hemodynamics was observed in the short term other than total pulmonary resistance, so further investigation is needed (NCT00469027). These studies using cell therapy are important for addressing the controversial question of the feasibility of patient-derived cellular phenotypes of PAH and for exploring the prospects of new treatment advances, such as using pulmonary organoids for drug screening in diverse conditions. These studies further promote a better understanding of rare pulmonary vascular diseases.

### 4.2 Auxiliary devices

CardioMEMS: The mortality of severe PAH with RHF is high. Are there any supporting devices available for PAH patients with decompensated RHF in addition to conventional therapy? The CHAMPION trial [111] found that maintaining pulmonary arterial pressure at a range of 10-25 mmHg in heart failure using CardioMEMS was promising. This study also found that the rehospitalization rate of HFrEF patients was reduced by 50% over a duration of 17.6 years. However, the increased adverse effects reported, such as injury of the pulmonary artery and hemoptysis, should be addressed by improving the inclusion criteria and operation technology to reduce the incidence of complications in the future.

Extracorporeal membrane oxygenation (ECMO): Patients will be recommended to the heart-lung transplant (HLTx) center for further treatment if they are defined as intermediate or high risk. ECMO is suggested for PAH patients after lung transplantation to minimize primary graft dysfunction or for patients with advanced PAH to prepare them for lung transplantation. A small-sample study [112] found that the 1-year survival rates of severe PH after lung transplantation exceeded 96%. However, evidence showed that immediate perioperative transplant survival was poor compared with that in other conditions. Implanted pumpless membrane oxygenators between the pulmonary artery and the pulmonary veins or left atrium (PA-LA) are another frequently used method to support the right ventricle.

### 4.3 Interventional therapies

For patients who are ineligible for lung transplantation, an extracorporeal life support (ECLS) strategy may be administered as an alternative therapy to help patients endure imminent conditions before transplantation [113]. The trans-catheter potts shunt [114] may be an appropriate option to decrease the load returning to the right side of the heart and thus support cardiopulmonary function. Other interventional therapies, such as atrial septostomy (AS) and balloon atrial septostomy (BAS), can be attempted only to palliate and bridge the interval to lung transplant and only in experienced heart centers.

Pulmonary artery denervation (PADN) is a novel technique that provides a promising therapeutic option for patients with PAH, as it can inhibit sympathetic stimulation [115]. A clinical trial is in progress (NCT02284737) to observe the changes in 6MWD and in hemodynamic responses.

## 5. Conclusion

PAH remains a life-threatening disease with a poor diagnosis despite the emergence of vasodilator therapies. Potential therapeutic targets of PAH are emerging with the growing understanding of pathological mechanisms. Therapeutic targets that focus on *BMPR2* mutations, apoptosis of PAECs and proliferation of PASMCs, inflammation, epigenetic modifications, and metabolic pathways will attract increasing attention in the near future. However, potential therapies require further investigation before implementation. Not every drug has the same efficacy for all patients. Different treatment approaches may potentially improve overall survival depending upon the distinct pathogenesis of the disease. The ongoing preclinical trials might offer new insight into therapeutic interventions in patients with PAH and additional rare diseases.
